# Racial/Ethnic Disparities in Post-acute Sequelae of SARS-CoV-2 Infection in New York: an EHR-Based Cohort Study from the RECOVER Program

**DOI:** 10.1007/s11606-022-07997-1

**Published:** 2023-02-16

**Authors:** Dhruv Khullar, Yongkang Zhang, Chengxi Zang, Zhenxing Xu, Fei Wang, Mark G. Weiner, Thomas W. Carton, Russell L. Rothman, Jason P. Block, Rainu Kaushal

**Affiliations:** 1 Department of Population Health Sciences, Weill Cornell Medicine, New York, NY USA; 2 Department of Medicine, Weill Cornell Medicine, New York, NY USA; 3 Louisiana Public Health Institute, New Orleans, LA USA; 4 Institute for Medicine and Public Health, Vanderbilt University Medical Center, Nashville, TN USA; 5 Department of Population Medicine, Harvard Pilgrim Health Care Institute, Harvard Medical School, Boston, MA USA

## Abstract

**Background:**

Compared to white individuals, Black and Hispanic individuals have higher rates of COVID-19 hospitalization and death. Less is known about racial/ethnic differences in post-acute sequelae of SARS-CoV-2 infection (PASC).

**Objective:**

Examine racial/ethnic differences in potential PASC symptoms and conditions among hospitalized and non-hospitalized COVID-19 patients.

**Design:**

Retrospective cohort study using data from electronic health records.

**Participants:**

62,339 patients with COVID-19 and 247,881 patients without COVID-19 in New York City between March 2020 and October 2021.

**Main Measures:**

New symptoms and conditions 31–180 days after COVID-19 diagnosis.

**Key Results:**

The final study population included 29,331 white patients (47.1%), 12,638 Black patients (20.3%), and 20,370 Hispanic patients (32.7%) diagnosed with COVID-19. After adjusting for confounders, significant racial/ethnic differences in incident symptoms and conditions existed among both hospitalized and non-hospitalized patients. For example, 31–180 days after a positive SARS-CoV-2 test, hospitalized Black patients had higher odds of being diagnosed with diabetes (adjusted odds ratio [OR]: 1.96, 95% confidence interval [CI]: 1.50—2.56, *q*<0.001) and headaches (OR: 1.52, 95% CI: 1.11—2.08, *q*=0.02), compared to hospitalized white patients. Hospitalized Hispanic patients had higher odds of headaches (OR: 1.62, 95% CI: 1.21—2.17, *q*=0.003) and dyspnea (OR: 1.22, 95% CI: 1.05—1.42, *q*=0.02), compared to hospitalized white patients. Among non-hospitalized patients, Black patients had higher odds of being diagnosed with pulmonary embolism (OR: 1.68, 95% CI: 1.20—2.36, *q*=0.009) and diabetes (OR: 2.13, 95% CI: 1.75—2.58, *q*<0.001), but lower odds of encephalopathy (OR: 0.58, 95% CI: 0.45—0.75, *q*<0.001), compared to white patients. Hispanic patients had higher odds of being diagnosed with headaches (OR: 1.41, 95% CI: 1.24—1.60, *q*<0.001) and chest pain (OR: 1.50, 95% CI: 1.35—1.67, *q* < 0.001), but lower odds of encephalopathy (OR: 0.64, 95% CI: 0.51—0.80, *q*<0.001).

**Conclusions:**

Compared to white patients, patients from racial/ethnic minority groups had significantly different odds of developing potential PASC symptoms and conditions. Future research should examine the reasons for these differences.

**Supplementary Information:**

The online version contains supplementary material available at 10.1007/s11606-022-07997-1.

## INTRODUCTION

Post-acute sequelae of SARS-CoV-2 infection (PASC) often refers to signs and symptoms that develop, persist, or worsen more than 30 days after acute infection with SARS-CoV-2.^1^ Research suggests that one in five patients may experience persistent symptoms or incident medical conditions after an acute COVID-19 infection, including dyspnea, fatigue, cognitive dysfunction, anxiety, and clotting disorders, among others.^2^ Several risk factors, such as age, pre-existing type 2 diabetes, and auto-antibodies, may increase the likelihood of developing PASC.^2,3^

Research suggests that Black and Hispanic individuals have higher rates of COVID-19 hospitalization and death compared to white individuals,^4^ but less is known about whether disparities exist with regard to the risk of developing new symptoms or conditions after acute COVID-19, or whether the prevalence of specific symptoms and conditions vary by racial/ethnic group. Compared to white individuals, racial/ethnic minority groups have differing levels of vaccination; access to medical care and COVID-19 therapeutics; baseline comorbidities; socioeconomic status; and viral exposure by virtue of their occupations and household structure—all of which may influence the risk of developing PASC.^5–10^

Studies examining potential racial/ethnic differences in PASC have yielded mixed results and generally focused on a single institution or specific patient populations (e.g., U.S. veterans or Medicare Advantage beneficiaries).^11–13^ Research examining the general adult population has focused on a limited set of potential PASC symptoms and conditions.^13^ In this study, we examined racial/ethnic differences in the incidence of potential PASC symptoms and conditions among a diverse cohort of hospitalized and non-hospitalized COVID-19 patients across five major academic institutions in New York City. We sought to answer three questions. First, what is the raw incidence of potential PASC symptoms and conditions among COVID-19 patients compared to patients not diagnosed with COVID-19? Second, among COVID-19 patients, does the raw incidence of new symptoms and conditions vary among racial/ethnic groups? Third, among COVID-19 patients, do the adjusted odds of developing a new symptom or condition differ for Black and Hispanic individuals compared to white individuals?

## METHODS

### Data Source, Setting, and Participants

We conducted a retrospective cohort study using electronic health record (EHR) data from the INSIGHT network, which collects data from five academic health systems in New York City.^14^ The COVID-19 database included 5,346,357 patients with one or more inpatient or outpatient visit for any reason within these health systems since January 1, 2020.

### Cohort Construction

We identified “COVID-19 positive” patients as those with a positive SARS-CoV-2 PCR/antigen test or COVID-19 diagnosis (U07.1, U07.2, J12.81, B34.2, B97.2, B97.21, U04, and U04.9) between March 1st, 2020, and October 31^st^, 2021. We included COVID-19-related diagnosis codes because some patients may have received a positive SARS-CoV-2 PCR/antigen tests outside INSIGHT health systems. We excluded patients with an “undetermined” result of a COVID-19 antigen/PCR test. We used the date of first PCR/antigen test or COVID-19 diagnosis as the index date. Patients were included if they were 20 years or older, had at least one encounter (i.e., visit) 3 years to 7 days before the index date (baseline period), and had at least one encounter 31–180 days after the index date (follow-up period). We defined COVID-19-negative patients as those with a negative PCR/antigen test, no positive PCR/antigen tests, or no COVID-19-related diagnosis codes during the same period. eFigure 1 presents the sample selection process.

### Defining PASC

In the absence of consensus about what constitutes PASC, we employed a pragmatic approach to define PASC signs and symptoms, which included review of the existing literature, input from clinical experts, and use of a data-driven analytic method.

First, we considered all ICD-10 diagnosis codes within the 540 diagnostic categories in the Clinical Classifications Software Refined (CCSR) version 2021.^15^ Based on clinician input and the existing literature,^12,16–20^ we identified diagnostic categories that could plausibly be considered PASC in the adult population. For example, diagnostic categories such as injury, trauma, poisoning, and other external causes of morbidity were excluded. The remaining diagnostic categories were reviewed and refined by a multidisciplinary team of physicians with expertise caring for COVID-19 patients. This resulted in a final list of 137 possible PASC diagnostic categories (eTable 1).

We then calculated the incidence of each potential PASC condition among the COVID-19-positive cohort versus the COVID-19-negative cohort using the following steps. First, for both COVID-19-positive and COVID-19-negative cohorts, we included patients without a diagnosis of a particular PASC condition during the baseline period. We examined diagnoses as outcomes separately and only excluded the specific diagnoses at baseline for models examining that same outcome (e.g., excluded patients with diabetes at baseline for analyses of the outcome of diabetes in the PASC period). These patients comprised the denominator from which we calculated the incidence of each PASC condition. Second, among patients without a given PASC diagnosis at baseline, we identified those with at least one diagnosis of that PASC condition during the follow-up period. These patients comprised the numerator for calculating the incidence. The incidence of each PASC condition was then calculated by dividing the number of patients in step 2 by the number of patients in step 3.

Finally, we used an inverse probability-weighted Cox proportional hazard model to compare the incidence risk of each of the 137 PASC conditions between COVID-19-positive and COVID-19-negative cohorts.^21^ We identified 24 PASC conditions with adjusted hazard ratios greater than 1 (meaning that COVID-19-positive patients were at higher risk of developing these conditions). These conditions were categorized into eight groups corresponding to organ systems (nervous, skin, respiratory, circulatory, blood, endocrine, digestive, and general), and were consistent with conditions reported in other studies of PASC.^1,11–13,22^

### Patient Characteristics

The key variable of interest was patient race/ethnicity. We used patient race/ethnicity data included in the EHR as specified by the Common Data Model of a national research network PCORnet, the National Patient-Centered Clinical Research Network,^23,24^ and categorized patients into three racial/ethnic groups: non-Hispanic White, non-Hispanic Black, and Hispanic. Patients from other racial/ethnic groups were not included, given small sample sizes.

We examined a comprehensive set of patient characteristics as potential confounders. These included patient age (20–39 [ref.], 40–54, 55–64, 65–74, 75–84, and 85+); gender (female [ref.], male, and other/missing); year-month of COVID-19 diagnosis (March 2020—October 2021); comorbidities; and indicators for the five institutions contributing data. As comorbidity covariates, we used a revised list of Elixhauser comorbidities, including alcohol abuse, anemia, arrythmia, asthma, cancer, chronic kidney disease, chronic pulmonary disorders, cirrhosis, coagulopathy, congestive heart failure, COPD, coronary artery disease, dementia, type-1 diabetes, type-2 diabetes, end-stage renal disease on dialysis, hemiplegia, HIV, hypertension, hypertension and type 1 or 2 diabetes diagnosis, inflammatory bowel disorder, lupus or systemic lupus erythematosus, mental health disorders, multiple sclerosis, Parkinson’s disease, peripheral vascular disorders, pregnant, pulmonary circulation disorder, rheumatoid arthritis, seizure/epilepsy, severe obesity (BMI≥40 kg/m^2^), and weight loss.^21^ Each comorbidity was identified using ICD-10 diagnosis codes.

Finally, because of the different disease trajectories, we stratified COVID-19-positive patients based on hospitalization status during their acute illness. Hospitalized patients were defined as those with an emergency department to inpatient, inpatient, or observation stay encounter 1 day prior through 16 days following the index date.^22,25^

### Statistical Analysis

Using *t*-tests for continuous variables and *χ*^2^ tests for categorical variables, we compared unadjusted differences in incidence of new conditions and symptoms across non-Hispanic White, non-Hispanic Black, and Hispanic COVID-19 patients. We used logistic regression to compare the likelihood of developing new conditions and/or symptoms across racial/ethnic groups, adjusting for the confounders described above. We also examined outcomes grouped by organ system rather than individual conditions and symptoms. All analyses were stratified by hospitalization status. We used the false discovery rate-adjusted *p*-value (*q*-value) to adjust for multiple comparisons for the adjusted analysis (significance threshold, *q*<0.05).^26^

We conducted several sensitivity analyses to examine the robustness of the results. First, we conducted analyses for COVID-19 patients identified only by positive PCR/antigen test, because the date of COVID-19 diagnosis may not reflect the true COVID-19 infection date. Second, we conducted analyses excluding patients whose index dates occurred during the first COVID-19 wave (March—May 2020), as SARS-CoV-2 testing was limited during this period. Third, we conducted analyses to determine whether other social factors might mediate the relationship between race/ethnicity and potential PASC symptoms and conditions. We repeated our primary analysis on a subset of patients for whom residential five-digit zip code was available and adjusted for socioeconomic characteristics at the zip-code tabulation area (ZCTA) level. These included median household income, unemployment rate, uninsurance rate, percent of population employed as essential workers, percent of population with limited English proficiency, percent of population foreign born, percent of population with crowded living conditions (more than 1.5 persons per room), and percent of population living in the same home for the past year, extracted from the U.S. Census Bureau’s 2019 American Community Survey.^27^ We also adjusted for the availability of neighborhood green space, measured by the Normalized Difference Vegetation Index from NASA’s Moderate Resolution Imaging Spectroradiometer.^28,29^

This study was part of the NIH RECOVER Initiative,^30^ which seeks to understand, treat, and prevent PASC, and was approved by the institutional review board at Weill Cornell Medicine (21-10-95-380).

## RESULTS

The study population included 29,331 White patients (47.1%; 4526 hospitalized, 24,805 non-hospitalized), 12,638 Black patients (20.3%; 3431 hospitalized, 9207 non-hospitalized), and 20,370 Hispanic patients (32.7%; 5149 hospitalized, 15,221 non-hospitalized) diagnosed with COVID-19 between March 2020 through October 2021. Among these patients, 59.7% were female; 29.2% had an index date between March and June 2020; and 34.9% had an index date between March and June 2021. Baseline comorbidities by race/ethnicity and hospitalization status are presented in Table 1.

**Table 1:** Baseline Characteristics of COVID-19-Positive Patients, by Race/Ethnicity and Hospitalization Status

Demographics and baseline comorbidities	All (*N* = 62,339)	White		Black		Hispanic	
		Hospitalized (*N* = 4526)	Non-hospitalized (*N* = 24,805)	Hospitalized (*N* = 3431)	Non-hospitalized (*N* = 9207)	Hospitalized (*N* = 5149)	Non-hospitalized (*N* = 15,221)
Demographics							
Age categories (%)							
20 to <40 years	22.9	11.9	22.0	14.3	25.4	19.9	29.1
40 to <55 years	23.7	12.2	22.6	18.0	30.0	18.0	28.5
55 to <65 years	21.6	17.8	22.1	25.8	23.6	19.4	20.5
65 to <75 years	18.0	25.2	19.6	22.4	13.8	21.0	13.5
75 to <85 years	10.2	20.4	10.5	14.5	5.9	15.2	6.5
85+ years	3.6	12.4	3.2	4.9	1.3	6.6	1.9
Race/ethnicity (%)							
Non-Hispanic white	47.1	–	–	–	–	–	–
Non-Hispanic black	20.3	–	–	–	–	–	–
Hispanic	32.7	–	–	–	–	–	–
Sex (%)							
Female	59.7	48.5	55.0	58.0	67.9	57.6	66.9
Male	40.3	51.5	45.0	42.0	32.2	42.4	33.1
Other/missing	0.0	0.0	0.0	0.0	0.0	0.0	0.0
Index date (%)							
March 2020–June 2020	29.2	31.9	25.1	42.0	33.0	39.8	26.5
July 2020–October 2020	14.9	11.7	18.0	7.0	14.4	8.6	15.0
November 2020–February 2021	34.9	36.5	36.9	28.2	29.9	31.9	36.7
March 2021–June 2021	16.4	15.5	15.1	18.0	17.3	16.4	18.0
July 2021–October 2021	4.6	4.4	5.0	4.8	5.6	3.3	3.8
Comorbidities, (%)							
Alcohol abuse	2.0	3.5	0.8	4.7	1.6	5.1	1.9
Anemia	10.8	18.4	5.8	28.0	12.2	20.8	8.5
Arrythmia	12.9	35.4	11.1	24.3	8.1	21.2	6.7
Asthma	10.2	8.7	6.1	16.4	11.3	17.2	13.0
Cancer	9.4	15.5	9.3	12.0	8.1	10.4	7.7
Chronic kidney disease	9.6	20.7	4.2	31.7	8.7	22.9	6.1
Chronic pulmonary disorders	15.1	20.2	11.0	25.6	14.5	23.3	15.4
Cirrhosis	1.2	2.1	0.7	1.9	0.6	3.3	1.3
Coagulopathy	4.7	16.0	2.6	11.1	2.4	10.8	2.6
Congestive heart failure	7.9	18.1	4.2	22.4	6.6	17.4	5.1
COPD	4.2	10.4	3.2	10.6	2.7	8.1	2.2
Coronary artery disease	12.3	27.4	11.3	20.8	7.1	21.4	7.6
Dementia	2.1	8.3	0.7	5.7	0.7	5.9	1.1
Diabetes type 1	1.0	1.3	0.7	2.0	0.7	2.1	0.8
Diabetes type 2	18.0	23.2	9.4	40.0	19.0	38.0	18.0
End-stage renal disease on dialysis	3.0	3.9	0.7	12.7	3.4	8.7	2.0
Hemiplegia	0.9	1.9	0.4	3.6	0.6	1.8	0.5
HIV	1.6	0.6	0.7	3.6	3.0	2.0	2.1
Hypertension	37.3	52.9	29.5	63.8	40.4	54.5	31.8
Hypertension and type 1 or 2 diabetes diagnosis	14.6	20.8	7.4	36.0	15.1	32.5	13.5
Inflammatory bowel disorder	1.2	1.9	1.7	1.3	0.5	0.8	0.7
Lupus or systemic lupus erythematosus	0.8	0.5	0.3	1.9	1.2	1.3	0.9
Mental health disorders	8.9	15.9	5.4	14.7	6.6	16.8	9.9
Multiple sclerosis	0.6	1.0	0.6	0.6	0.8	0.4	0.4
Parkinson’s disease	0.5	1.9	0.5	0.8	0.2	1.0	0.3
Peripheral vascular disorders	6.1	12.9	4.2	13.8	4.6	12.4	4.2
Pregnant	3.3	6.0	1.6	4.0	2.3	9.3	3.6
Pulmonary circulation disorder	1.3	3.2	0.8	4.1	1.4	2.0	0.7
Rheumatoid arthritis	1.5	1.8	1.2	2.5	1.3	2.0	1.8
Seizure/epilepsy	1.6	3.8	1.0	3.6	1.3	3.2	1.2
Severe obesity (BMI≥40 kg/m^2^)	6.6	7.4	3.6	13.6	9.1	9.2	7.3
Weight Loss	2.8	7.7	1.4	7.8	2.2	5.9	1.7

Table 2 presents the 24 incident symptoms or conditions (grouped into 8 categories by organ system) identified. Compared to COVID-19-negative patients, COVID-19 patients had higher levels of nervous system disorders (10.3% vs 7.6%, *p*<0.001), respiratory conditions (13.4% vs 7.3%, *p*<0.001), circulatory system conditions (10.6% vs 7.1%, *p*<0.001), and general signs and symptoms, such as fatigue and joint pain (13.4% vs 10.6%, *p*<0.001) 31–180 days after a positive SARS-CoV-2 test. Among COVID-19 patients, individuals who were hospitalized had higher rates of incident symptoms and conditions compared to those who were not hospitalized.

**Table 2:** Incidence of New Conditions and Symptoms Among COVID-19-Positive and COVID-19-Negative Patients, by COVID-19 and Hospitalization Status

	COVID-19 positive (%)			COVID-19 negative (%)		
	All (*N* = 62,339)	Hospitalized (*N* = 13,106)	Non-hospitalized (*N* = 49,233)	All (*N* = 247,881)	Hospitalized (*N* = 71,222)	Non-hospitalized (*N* = 176,659)
Nervous						
Encephalopathy	1.7	3.3	1.2	1.1	2.2	0.6
Dementia	0.9	3.1	0.4	0.8	2.1	0.4
Cognitive problems	3.8	6.9	3.1	2.7	4.7	2.0
Sleep disorders	3.7	4.8	3.4	2.7	2.9	2.7
Headache	3.5	3.2	3.6	2.4	2.3	2.4
Any nervous condition	10.3	14.8	9.2	7.6	10.2	6.6
Skin						
Hair loss	1.2	1.1	1.2	0.6	0.4	0.6
Pressure ulcer of skin	0.6	2.4	0.2	0.5	1.3	0.1
Any skin conditions	1.8	3.5	1.4	1.0	1.7	0.8
Respiratory						
Lower respiratory disease	2.7	5.3	2	1.1	1.5	0.9
Dyspnea	11.8	16.8	10.5	6.4	7.4	6.0
Upper respiratory infections	1.3	1.1	0.9	0.9	0.6	1.0
Any respiratory condition	13.4	18.9	11.9	7.3	8.1	6.9
Circulatory						
Pulmonary embolism	0.8	2.3	0.4	0.5	1.1	0.3
Thromboembolism	1.3	3.2	0.8	1.0	2.0	0.6
Chest pain	5.8	6.5	5.6	3.7	3.9	3.6
Abnormal heartbeat	5.3	7.6	4.7	3.3	4.6	2.9
Any circulatory condition	10.6	15.4	9.5	7.1	9.4	6.3
Blood						
Anemia	4.2	9.4	3.0	3.7	7.2	2.4
Endocrine						
Malnutrition	1.6	4.8	0.7	1.4	3.3	0.7
Diabetes mellitus	3.1	6.8	2.3	2.7	4.3	2.1
Fluid and electrolyte disorders	0.5	1.6	0.3	0.4	0.9	0.2
Edema	6.6	10.5	5.6	5.5	7.6	4.7
Any endocrine condition	9.4	17.6	7.8	8.1	11.8	6.7
Digestive						
Other constipation	3.7	6.3	3.0	3.6	4.9	3.1
Abdominal pain	8.4	10.1	8.0	7.3	8.3	6.9
Any digestive condition	10.2	13.8	9.3	9.0	10.9	8.2
General signed and symptoms						
Malaise and fatigue	4.9	7.8	4.2	3.3	4.6	2.8
Fever	0.2	0.5	0.1	0.2	0.3	0.1
Joint pain	10.0	10.8	9.8	8.1	8.2	8.0
Any general signed and symptoms	13.4	16.0	12.7	10.6	11.7	10.1

Notes: When calculating the incidence of each new condition and symptom, the denominator is the number of COVID-19-positive patients without any diagnosis of the condition or symptom at baseline and the numerator is the number of COVID-19 patients without the diagnosis at baseline but with at least one diagnosis of the condition or symptom in the follow-up period. *p* values indicate the statistical significance of differences in PASC incidence comparing COVID-19-positive and COVID-19-negative patients, using *χ*^2^ tests.

Table 3 shows unadjusted differences in incidence of symptoms and conditions by race/ethnicity and by hospitalization status among COVID-19 patients. Among hospitalized COVID-19 patients, several racial/ethnic differences in incident conditions were observed. For example, compared to hospitalized white COVID-19 patients, hospitalized Black COVID-19 patients had higher rates of incident circulatory system disorders (16.7% vs 13.8%, *p*=0.004), anemia (10.9% vs 9.0, *p*=0.01), endocrine conditions (19.4% vs 16.6%, *p*=0.02), and general signs and symptoms (17.0% vs 14.9%, *p*=0.04) 31–180 days after a positive SARS-CoV-2 test. Compared to white patients, hospitalized Hispanic COVID-19 patients had higher rates of incident circulatory system conditions (16.1% vs 13.8%, *p*=0.01) and digestive system conditions (15.9% vs 12.1%, *p*<0.001) but lower rates of incident nervous system conditions (16.2% vs 13.7%, *p*=0.006) and skin conditions (4.0 vs 3.1, *p*=0.02). Similar differences were observed among non-hospitalized COVID-19 patients, although the absolute rates of incident symptoms and conditions were lower.

**Table 3:** Incidence of New Conditions and Symptoms Among COVID-19-Positive Patients, by Race/Ethnicity

	Hospitalized COVID-19 patients (%)			Non-hospitalized COVID-19 patients (%)		
	Non-Hispanic White	Non-Hispanic Black	Hispanic	Non-Hispanic White	Non-Hispanic Black	Hispanic
Nervous						
Encephalopathy	4.1	3.2 (*p* = 0.03)	2.6 (*p* < 0.001)	1.5	1.0 (*p* = 0.001)	1.0 (*p* < 0.001)
Dementia	3.6	2.7 (*p* = 0.03)	2.9 (*p* = 0.04)	0.3	0.3 (*p* = 0.49)	0.6 (*p* < 0.001)
Cognitive problems	8.1	6.5 (*p* = 0.01)	6.1 (*p* < 0.001)	3.1	2.9 (*p* = 0.37)	3.2 (*p* = 0.33)
Sleep disorders	6.1	3.9 (*p* < 0.001)	4.4 (*p* < 0.001)	3.3	3.4 (*p* = 0.75)	3.5 (*p* = 0.38)
Headache	2.2	3.3 (*p* = 0.004)	3.9 (*p* < 0.001)	2.8	3.8 (*p* < 0.001)	4.8 (*p* < 0.001)
Any nervous condition	16.2	14.7 (*p* = 0.16)	13.7 (*p* = 0.006)	8.5	9.3 (*p* = 0.07)	10.3 (*p* < 0.001)
Skin						
Hair loss	1.2	0.7 (*p* = 0.01)	1.2 (*p* = 0.94)	1.1	0.9 (*p* = 0.04)	1.5 (*p* < 0.001)
Pressure ulcer of skin	2.8	2.6 (*p* = 0.61)	2.0 (*p* = 0.005)	0.1	0.2 (*p* = 0.04)	0.2 (*p* = 0.07)
Any skin conditions	4.0	3.3 (*p* = 0.09)	3.1 (*p* = 0.02)	1.2	1.1 (*p* = 0.22)	1.8 (*p* < 0.001)
Respiratory						
Lower respiratory disease	5.1	5.5 (*p* = 0.44)	5.4 (*p* = 0.57)	2.0	1.9 (*p* = 0.48)	2.0 (*p* = 0.88)
Dyspnea	16.3	16.8 (*p* = 0.64)	17.1 (*p* = 0.40)	10.1	10.2 (*p* = 0.97)	11.4 (*p* = 0.001)
Upper respiratory infections	1.1	1.1 (*p* = 0.91)	1.0 (*p* = 0.78)	1.2	1.4 (*p* = 0.23)	1.5 (*p* = 0.04)
Any respiratory condition	17.9	19.3 (*p* = 0.21)	19.4 (*p* = 0.12)	11.4	11.5 (*p* = 0.97)	13.0 (*p* < 0.001)
Circulatory						
Pulmonary embolism	2.3	3.0 (*p* = 0.06)	1.8 (*p* = 0.08)	0.4	0.7 (*p* = 0.001)	0.4 (*p* = 0.87)
Thromboembolism	3.4	3.4 (*p* = 0.96)	2.9 (*p* = 0.15)	0.7	1.0 (*p* = 0.02)	0.7 (*p* = 0.82)
Chest pain	4.6	7.4 (*p* < 0.001)	7.6 (*p* < 0.001)	4.6	6.4 (*p* < 0.001)	7.0 (*p* < 0.001)
Abnormal heartbeat	7.9	6.8 (*p* = 0.10)	7.8 (*p* = 0.89)	4.4	4.1 (*p* = 0.21)	5.4 (*p* < 0.001)
Any circulatory condition	13.8	16.7 (*p* = 0.004)	16.1 (*p* = 0.01)	8.4	10.0 (*p* < 0.001)	10.9 (*p* < 0.001)
Blood						
Anemia	9.0	10.9 (*p* = 0.01)	8.7 (*p* = 0.64)	2.5	4.0 (*p* < 0.001)	3.4 (*p* < 0.001)
Endocrine						
Malnutrition	5.9	4.6 (*p* = 0.01)	4.0 (*p* < 0.001)	0.5	1.0 (*p* < 0.001)	0.9 (*p* < 0.001)
Diabetes mellitus	4.6	8.0 (*p* < 0.001)	8.4 (*p* < 0.001)	1.6	3.1 (*p* < 0.001)	3.1 (*p* < 0.001)
Fluid and electrolyte disorders	1.5	1.8 (*p* = 0.34)	1.4 (*p* = 0.53)	0.2	0.3 (*p* = 0.45)	0.3 (*p* = 0.02)
Edema	11.3	10.6 (*p* = 0.40)	9.7 (*p* = 0.02)	5.3	6.1 (*p* = 0.01)	5.9 (*p* = 0.05)
Any endocrine condition	16.6	19.4 (*p* = 0.02)	17.1 (*p* = 0.32)	6.9	9.0 (*p* < 0.001)	8.6 (*p* < 0.001)
Digestive						
Other constipation	5.7	6.1 (*p* = 0.55)	6.8 (*p* = 0.04)	2.6	2.9 (*p* = 0.10)	3.7 (*p* < 0.001)
Abdominal pain	8.5	9.8 (*p* = 0.10)	11.9 (*p* < 0.001)	6.5	8.3 (*p* < 0.001)	10.4 (*p*< 0.001)
Any digestive condition	12.1	13.2 (*p* = 0.24)	15.9 (*p* < 0.001)	7.7	9.5 (*p* < 0.001)	12.1 (*p* < 0.001)
General signed and symptoms						
Malaise and fatigue	9.2	7.8 (*p* = 0.04)	6.5 (*p* < 0.001)	4.5	3.7 (*p* < 0.001)	3.9 (*p* = 0.01)
Fever	0.7	0.4 (*p* = 0.07)	0.5 (*p* = 0.12)	0.1	0.1 (*p* = 0.84)	0.2 (*p* = 0.005)
Joint pain	8.7	11.7 (*p* < 0.001)	12.2 (*p* < 0.001)	7.9	11.0 (*p* < 0.001)	12.4 (*p* < 0.001)
Any general signed and symptoms	14.9	17.0 (*p* = 0.04)	16.3 (*p* = 0.11)	11.0	13.7 (*p* < 0.001)	12.5 (*p* < 0.001)

**Figure 1:**
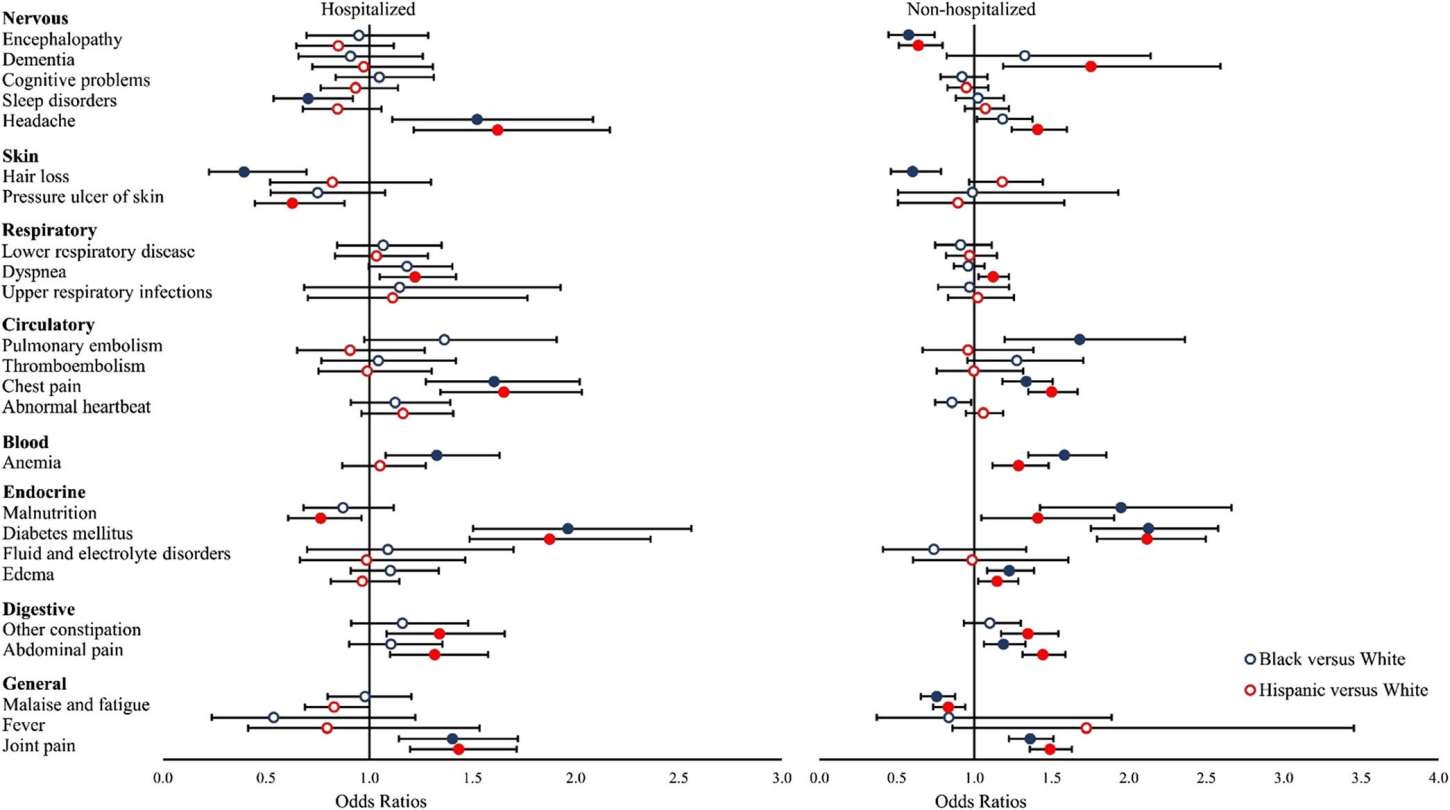
Adjusted differences in incidence of new conditions and symptoms by race/ethnicity among hospitalized and non-hospitalized COVID-19 patients. Notes: Odds ratios (ORs) were estimated from logistic regressions examining the outcome of having at least diagnosis code of each new condition and symptom category during the follow-up period (Reference group = White). Models were adjusted for baseline patient characteristics, including age, gender, year-month of COVID-19 positive testing, comorbidities, and indicators for the five institutions contributing data. * Filled symbols indicate significant ORs that are statistically significant after false discovery rate correction (q < 0.05).

Significant racial/ethnic differences persisted after adjustment for potential confounders for both hospitalized and non-hospitalized COVID-19 patients (Fig. 1). Compared to hospitalized white COVID-19 patients, hospitalized Black COVID-19 patients had significantly higher odds of being diagnosed with headaches (OR: 1.52, 95% confidence interval [CI]: 1.11—2.08, *q*=0.02), chest pain (OR: 1.61, 95% CI: 1.28—2.02, *q*<0.001), diabetes (OR: 1.96, 95% CI: 1.50—2.56, *q*<0.001), and joint pain (OR: 1.40, 95% CI: 1.14—1.72, *q*=0.003), but lower rates of sleep disorders (OR: 0.70, 95% CI: 0.54—0.92, *q*=0.03) and hair loss (OR: 0.39, 95% CI: 0.22—0.70, *q*=0.003) 31–180 days after a positive SARS-CoV-2 test. Compared to hospitalized white COVID-19 patients, hospitalized Hispanic COVID-19 patients had higher odds of developing headaches (OR: 1.62, 95% CI: 1.21—2.17, *q*=0.003), dyspnea (OR: 1.22, 95% CI: 1.05—1.42, *q*=0.02), chest pain (OR: 1.65, 95% CI: 1.35—2.03, *q*<0.001), and joint pain (OR: 1.43, 95% CI: 1.20—1.72, *q*<0.001), but lower odds of pressure ulcers (OR: 0.63, 95% CI: 0.45—0.88, *q*=0.02) and malnutrition (OR: 0.76, 95% CI: 0.61—0.96, *q*=0.048).

Non-hospitalized COVID-19 patients demonstrated distinct adjusted racial/differences (Fig. 1). For example, compared to non-hospitalized white COVID-19 patients, non-hospitalized Black COVID-19 patients had higher odds of developing pulmonary embolism (OR: 1.68, 95% CI: 1.20—2.36, *q*=0.009), chest pain (OR: 1.34, 95% CI: 1.18—1.51, *q*<0.001), anemia (OR: 1.58, 95% CI: 1.35—1.85, *q*<0.001), malnutrition (OR: 1.95, 95% CI: 1.43—2.66, *q*<0.001), diabetes (OR: 2.13, 95% CI: 1.75—2.58, *q*<0.001), and joint pain (OR: 1.36, 95% CI: 1.23—1.51, *q*<0.001), but lower odds of encephalopathy (OR: 0.58, 95% CI: 0.45—0.75, *q*<0.001), hair loss (OR: 0.60, 95% CI: 0.46—0.79, *q*<0.001), and malaise/fatigue (OR: 0.76, 95% CI: 0.66—0.88, *q*<0.001) 31–180 days after a positive SARS-CoV-2 test. Non-hospitalized Hispanic COVID-19 patients had higher odds of being diagnosed with dementia (OR: 1.75, 95% CI: 1.19—2.59, *q*=0.01), headache (OR: 1.41, 95% CI: 1.24—1.60, *q*<0.001), chest pain (OR: 1.50, 95% CI: 1.35—1.67, *q*<0.001), anemia (OR: 1.29, 95% CI: 1.12—1.48, *q*=0.002), and diabetes (OR: 2.12, 95% CI: 1.79—2.50, *q*<0.001), among others. They had lower odds of being diagnosed with encephalopathy (OR: 0.64, 95% CI: 0.51—0.80, *q*<0.001) and malaise/fatigue (OR: 0.83, 95% CI: 0.74—0.94, *q*=0.009).

**Figure 2:**
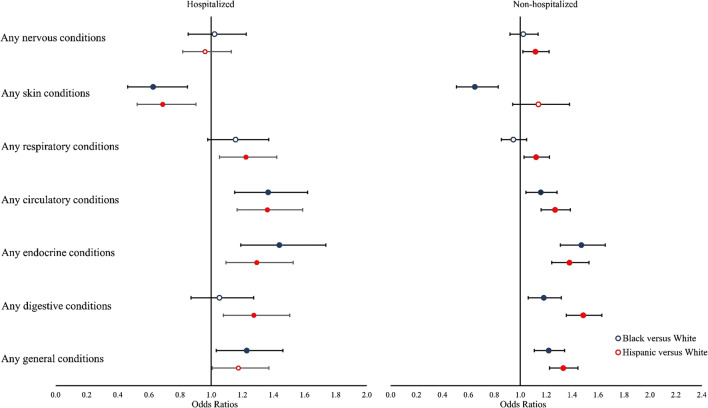
Adjusted differences in incidence of groups conditions and symptoms by race/ethnicity among hospitalized and non-hospitalized COVID-19 patients. Notes: Odds ratios (ORs) were estimated from logistic regressions examining the outcome of having at least one condition or symptom in each group during the follow-up period (Reference group = White). Models were adjusted for baseline patient characteristics, including age, gender, year-month of COVID-19 positive testing, comorbidities, and indicators for the five institutions contributing data. * Filled symbols indicate significant ORs that are statistically significant after false discovery rate correction (q < 0.05).

Figure 2 shows adjusted racial/ethnic differences in incident symptoms and conditions grouped by organ system. Compared to hospitalized white COVID-19 patients, hospitalized Black COVID-19 patients had higher odds of circulatory system conditions (OR: 1.37, 95% CI: 1.15—1.62, *q*=0.001), endocrine conditions (OR: 1.44, 95% CI: 1.19—1.74, *q*<0.001), and general signs and symptoms (OR: 1.23, 95% CI: 1.03—1.46, *q*=0.04) 31–180 days after a positive SARS-CoV-2 test; hospitalized Hispanic COVID-19 patients had higher odds of respiratory conditions (OR: 1.22, 95% CI: 1.05—1.42, *q*=0.02), circulatory system conditions (OR: 1.36, 95% CI: 1.17—1.59, *q*<0.001), endocrine conditions (OR: 1.29, 95% CI: 1.09—1.53, *q*=0.009), and digestive system conditions (OR: 1.27, 95% CI: 1.08—1.50, *q*=0.001). Results were similar among non-hospitalized COVID-19 patients.

### Sensitivity Analysis

We found similar results when analyzing only patients identified based on positive SARS-CoV-2 PCR/antigen testing (eFigures 2 and 3). In the analysis excluding COVID-19 patients from the first wave of the pandemic (March—May 2020), disparities among hospitalized patients diminished among some PASC conditions and symptoms, although significant racial/ethnicity disparities were consistent among non-hospitalized patients (eFigures 4 and 5). In analyses adjusting for neighborhood-level socioeconomic characteristics, significant differences in potential PASC symptoms and conditions persisted among both hospitalized and non-hospitalized patients, though several differences no longer reached significance (eFigures 6 and 7).

## DISCUSSION

We present novel evidence on racial/ethnic differences in the potential long-term consequences of SARS-CoV-2 infection for hospitalized and non-hospitalized COVID-19 patients in New York City. We find that the incidence of potential PASC symptoms and conditions differed significantly across racial/ethnic groups, and that compared to white patients, patients from racial/ethnic minority groups had significantly higher adjusted odds of a being diagnosed with a range of symptoms and conditions affecting multiple organ systems. For example, among non-hospitalized COVID-19 patients, Hispanic individuals had higher adjusted odds of being diagnosed with potential PASC across 6 of 8 organ systems; Black individuals had higher odds of diagnosis across 4 of 8 systems. Among hospitalized patients, adjusted differences were especially pronounced for endocrine and circulatory system conditions. For example, compared to white patients hospitalized with COVID-19, Black patients had approximately twice the odds of being diagnosed with diabetes, and one-and-a-half times the odds of being diagnosed with chest pain in the 30–180 days after infection. Similar differences were observed for Hispanic individuals.

The reasons for these differences are not clear. There are several possible contributors. Prior research suggests that Black and Hispanic individuals are more likely to experience severe acute COVID-19, which may increase the risk of PASC.^31^ Simple stratifications by care setting (e.g., hospitalized vs. non-hospitalized) may not adequately account for differences in disease severity within care settings. These populations may also experience higher risk of viral exposure and poorer access to timely medical care, which may lead to more severe acute illness and more long-term sequelae.^32,33^ Particularly for hospitalized COVID-19 patients, racial/ethnic disparities in the availability and quality of post-acute care may influence the development of PASC symptoms and conditions.^34,35^ In the months after COVID-19 vaccines became available, racial/ethnic disparities existed in rates of COVID-19 immunization, which may affect rates of PASC (these differences narrowed by the end of our study period).^36^ Finally, racial/ethnic minority groups may have lower baseline health status and socioeconomic conditions,^37^ placing them at higher risk for long-term complications. In sensitivity analyses examining neighborhood-level socioeconomic characteristics, significant differences in potential PASC symptoms and conditions largely remained, although in some cases were attenuated. This suggests that while factors such as housing security, living conditions, and the stability and type of an individual’s employment may partially mediate the relationship between race/ethnicity and PASC, race/ethnicity may be an independent risk factor for experiencing lasting health effects after acute COVID-19.

Our results suggest that racial/ethnic minority groups may experience a higher overall burden of potential PASC after COVID-19 compared to white individuals, but also that they may differ in the types of symptoms and conditions they experience. Understanding and addressing these differences is important as research on treatment for PASC continues, and physicians, funders, and health systems aim to address the long-term consequences of COVID-19 for all patient populations. Our study suggests that different communities may have different experiences and needs, and that ensuring diversity of enrollment in PASC trials and clinics is essential.

Strengths of this study include a large cohort of COVID-19 patients from the general adult population; examination of a broad set of potential PASC symptoms and conditions identified from a rigorous causal inference technique; and use of longitudinal data for comprehensive patient clinical encounters and characteristics before and after acute COVID-19 infection.

This study also has limitations. Similar to other work,^11^ we identified PASC incidence using single diagnosis codes, which are more reliable for measuring clinical conditions than patient symptoms and do not capture patients who do not seek care. These codes represent those entered into EHRs, and some diagnoses may reflect receipt of more medical care after an acute illness instead of long-term consequences of SARS-CoV-2 infection. It is also possible that some incident conditions represent preexisting conditions that were not diagnosed before the index COVID-19 illness. Furthermore, we controlled for a broad array of demographic and clinical variables, but residual confounding may occur. Finally, our analysis was conducted in an urban population, and may not generalize to other settings.

In this study, we leveraged EHR data from five health systems across New York City to analyze possible PASC in a large and demographically diverse patient population. We found significant racial/ethnic differences in the rates of new symptoms and conditions for both hospitalized and non-hospitalized COVID-19, after adjusting for a comprehensive set of possible confounders. Future research should examine the reasons for these differences, including patient, health system, and socioeconomic factors, and whether similar disparities are present in other regions of the USA.

## Supplementary Information

ESM 1 (DOCX 1437 kb)
