# Supplementary material for: Racial/Ethnic Disparities in Post-acute Sequelae of SARS-CoV-2 Infection in New York: an EHR-Based Cohort Study from the RECOVER Program
Source: J Gen Intern Med. 2023 Feb 16;38(5):1127–36. doi: 10.1007/s11606-022-07997-1 (PMC9933823; doi:10.1007/s11606-022-07997-1)
Supplement: Supplementary file 1 — (DOCX 1437 kb) [file 11606_2022_7997_MOESM1_ESM.docx]

**SUPPLEMENTAL ONLINE CONTENT**

**Racial/Ethnic Disparities in Post-Acute Sequelae of SARS-CoV-2 Infection in New York: An EHR-based Cohort Study from the RECOVER Program**

**eFigure 1.** Flow Chart of Sample Selection

**eTable 1.** List of 137 PASC Categories

**eFigure 2.** Adjusted Differences in Incidence of New Conditions and Symptoms by Race/Ethnicity Among Hospitalized and Non-Hospitalized COVID-19 Patients, Patients with a Positive PCR/Antigen Test Only

**eFigure 3.** Adjusted Differences in Incidence of Groups of Conditions and Symptoms by Race/Ethnicity Among Hospitalized and Non-Hospitalized COVID-19 Patients, Patients with a Positive PCR/Antigen Test Only

**eFigure 4.** Adjusted Differences in Incidence of New Conditions and Symptoms by Race/Ethnicity Among Hospitalized and Non-Hospitalized COVID-19 Patients, Excluding COVID-19 Patients in the First Wave of the Pandemic (March through May 2020) in NYC

**eFigure 5.** Adjusted Differences in Incidence of Groups of Conditions and Symptoms by Race/Ethnicity Among Hospitalized and Non-Hospitalized COVID-19 Patients, Excluding COVID-19 Patients in the First Wave of the Pandemic (March through May 2020) in NYC

**eFigure 6.** Adjusted Differences in Incidence of New Conditions and Symptoms by Race/Ethnicity Among Hospitalized COVID-19 Patients, Analysis Adding Neighborhood Social Conditions

**eFigure 7.** Adjusted Differences in Incidence of New Conditions and Symptoms by Race/Ethnicity Among Non-Hospitalized COVID-19 Patients, Analysis Adding Neighborhood Social Conditions

This supplementary material has been provided by the authors to give readers additional

information about their work.

**eFigure 1. Flow Chart of Sample Selection**


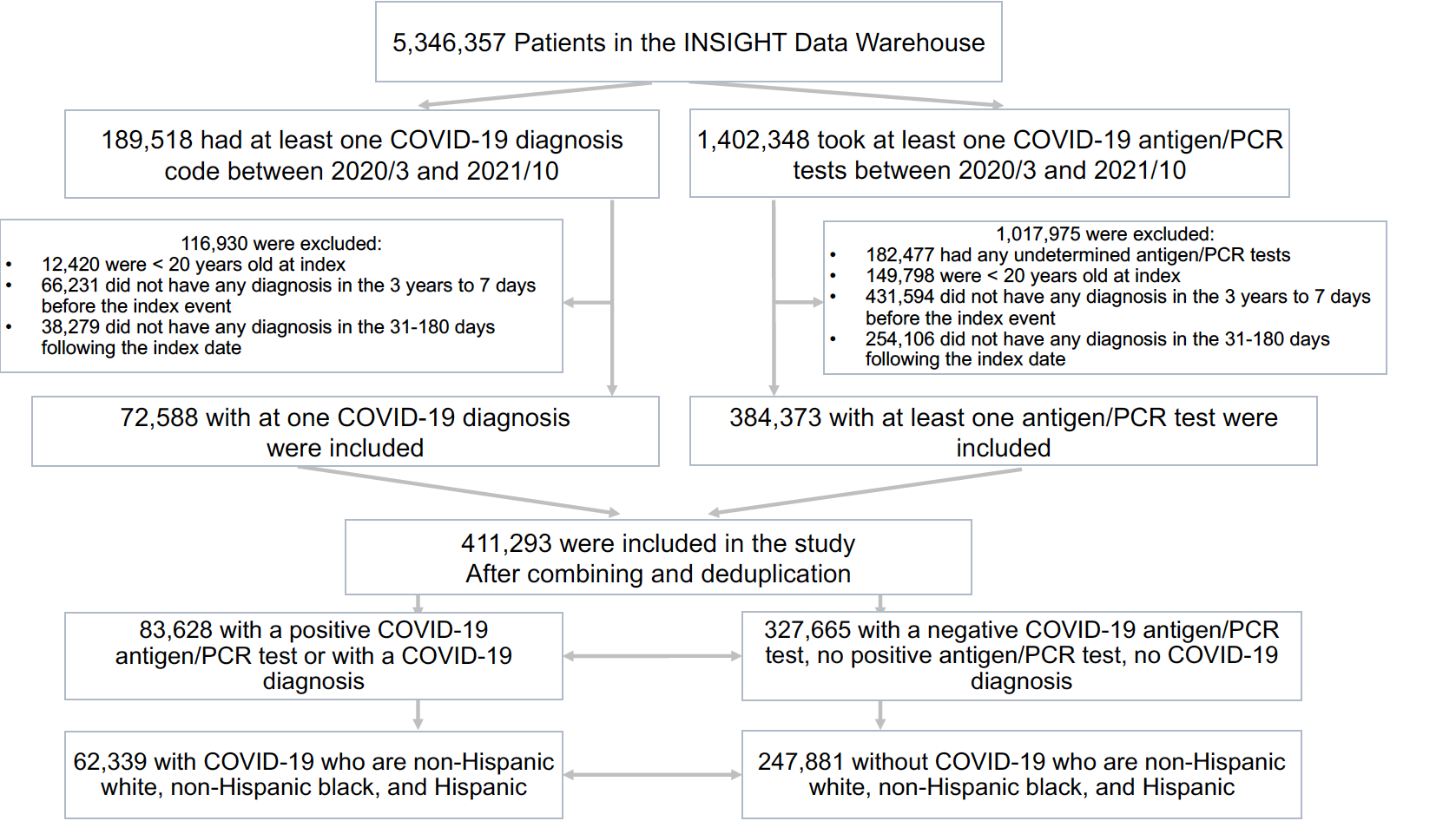


**eTable 1. List of 137 PASC Categories**

| ID | PASC Categories^1^ | Corresponding CCSR Categories^2^ |
| --- | --- | --- |
|  | Abdominal pain | Abdominal pain and other digestive/abdomen signs and symptoms |
|  | Abnormal findings related to substance use | Abnormal findings related to substance use |
|  | Abnormal heartbeat | Circulatory signs and symptoms |
|  | Acquired deformities (excluding foot) | Acquired deformities (excluding foot) |
|  | Acute and chronic tonsillitis | Acute and chronic tonsillitis |
|  | Acute bronchitis | Acute bronchitis |
|  | Acute kidney failure | Acute and unspecified renal failure |
|  | Acute myocardial infarction | Acute myocardial infarction |
|  | Alcohol-related disorders | Alcohol-related disorders |
|  | Allergy | Allergic reactions, subsequent encounter |
|  | Anemia | Anemia |
|  | Anxiety and fear-related disorders | Anxiety and fear-related disorders |
|  | Aortic and peripheral arterial embolism or thrombosis | Aortic and peripheral arterial embolism or thrombosis |
|  | Arterial dissections | Arterial dissections |
|  | Aseptic necrosis and osteonecrosis | Aseptic necrosis and osteonecrosis |
|  | Aspiration pneumonitis | Aspiration pneumonitis |
|  | Asthma | Asthma |
|  | Atelectasis | Pleurisy, pleural effusion and pulmonary collapse |
|  | Autoinflammatory syndromes | Autoinflammatory syndromes |
|  | Biliary tract disease | Biliary tract disease |
|  | Bipolar and related disorders | Bipolar and related disorders |
|  | Cannabis-related disorders | Cannabis-related disorders |
|  | Cardiac arrest and ventricular fibrillation | Cardiac arrest and ventricular fibrillation |
|  | Cardiac dysrhythmias | Cardiac dysrhythmias |
|  | Cerebral infarction | Cerebral infarction |
|  | Cerebral ischemia | Other and ill-defined cerebrovascular disease |
|  | Chest pain | Nonspecific chest pain |
|  | Cocaine abuse | Stimulant-related disorders |
|  | Cognitive problems | Nervous system signs and symptoms |
|  | Coma; stupor; and brain damage | Coma; stupor; and brain damage |
|  | Constipation | Other specified and unspecified gastrointestinal disorders |
|  | COPD | Chronic obstructive pulmonary disease and bronchiectasis |
|  | Coronary atherosclerosis and other heart disease | Coronary atherosclerosis and other heart disease |
|  | Crystal arthropathies (excluding gout) | Crystal arthropathies (excluding gout) |
|  | Cystitis | Urinary tract infections |
|  | Dementia | Neurocognitive disorders |
|  | Depressive disorders | Depressive disorders |
|  | Dermatitis | Allergic reactions |
|  | Diabetes mellitus | Diabetes mellitus with complication |
|  | Diabetes mellitus without complication | Diabetes mellitus without complication |
|  | Diseases of inner ear and related conditions | Diseases of inner ear and related conditions |
|  | Diseases of the Genitourinary System | Diseases of the Genitourinary System |
|  | Dizziness | General sensation/perception signs and symptoms |
|  | Drug induced or toxic related condition | Drug induced or toxic related condition |
|  | Dyspnea | Respiratory signs and symptoms |
|  | Edema | Other general signs and symptoms |
|  | Encephalitis | Encephalitis |
|  | Encephalopathy | Other nervous system disorders (neither hereditary nor degenerative) |
|  | Epilepsy; convulsions | Epilepsy; convulsions |
|  | Exposure, encounters, screening or contact with infectious disease | Exposure, encounters, screening or contact with infectious disease |
|  | Feeding and eating disorders | Feeding and eating disorders |
|  | Fever | Fever |
|  | Fibromyalgia | Other specified connective tissue disease |
|  | Fluid disorders | Fluid and electrolyte disorders |
|  | Foot drop | Acquired foot deformities |
|  | Gangrene | Gangrene |
|  | Gastritis and duodenitis | Gastritis and duodenitis |
|  | Gastroduodenal ulcer | Gastroduodenal ulcer |
|  | Gastroparesis | Other specified and unspecified disorders of stomach and duodenum |
|  | Genitourinary symptoms | Genitourinary signs and symptoms |
|  | GERD | Esophageal disorders |
|  | Gout | Gout |
|  | Hair loss | Other specified and unspecified skin disorders |
|  | Hallucinogen-related disorders | Hallucinogen-related disorders |
|  | Headache | Headache; including migraine |
|  | Hearing loss | Hearing loss |
|  | Heart failure | Heart failure |
|  | Hepatic failure | Hepatic failure |
|  | Hypotension | Hypotension |
|  | Immune-mediated/reactive arthropathies | Immune-mediated/reactive arthropathies |
|  | Intestinal obstruction and ileus | Intestinal obstruction and ileus |
|  | Joint pain | Musculoskeletal pain, not low back pain |
|  | Lower respiratory disease | Other specified and unspecified lower respiratory disease |
|  | Malaise and fatigue | Malaise and fatigue |
|  | Malnutrition | Malnutrition |
|  | Mediastinal disorders | Mediastinal disorders |
|  | Miscellaneous mental and behavioral disorders/conditions | Miscellaneous mental and behavioral disorders/conditions |
|  | Muscle weakness | Muscle disorders |
|  | Myocarditis and cardiomyopathy | Myocarditis and cardiomyopathy |
|  | Myopathies | Myopathies |
|  | Nausea and vomiting | Nausea and vomiting |
|  | Nephritis; nephrosis; renal sclerosis | Nephritis; nephrosis; renal sclerosis |
|  | Nerve and nerve root disorders | Nerve and nerve root disorders |
|  | Nervous system pain and pain syndromes | Nervous system pain and pain syndromes |
|  | Neurodevelopmental disorders | Neurodevelopmental disorders |
|  | Nicotine dependence | Tobacco-related disorders |
|  | Noninfectious hepatitis | Noninfectious hepatitis |
|  | Obsessive-compulsive and related disorders | Obsessive-compulsive and related disorders |
|  | Occlusion or stenosis of precerebral or cerebral arteries without infarction | Occlusion or stenosis of precerebral or cerebral arteries without infarction |
|  | Opioid-related disorders | Opioid-related disorders |
|  | Osteoarthritis | Osteoarthritis |
|  | Other and ill-defined heart disease | Other and ill-defined heart disease |
|  | Other nervous system disorders (often hereditary or degenerative) | Other nervous system disorders (often hereditary or degenerative) |
|  | Other specified and unspecified circulatory disease | Other specified and unspecified circulatory disease |
|  | Other specified and unspecified diseases of kidney and ureters | Other specified and unspecified diseases of kidney and ureters |
|  | Other specified and unspecified disorders of the ear | Other specified and unspecified disorders of the ear |
|  | Other specified and unspecified liver disease | Other specified and unspecified liver disease |
|  | Other specified and unspecified mood disorders | Other specified and unspecified mood disorders |
|  | Other specified bone disease and musculoskeletal deformities | Other specified bone disease and musculoskeletal deformities |
|  | Other specified inflammatory condition of skin | Other specified inflammatory condition of skin |
|  | Other specified joint disorders | Other specified joint disorders |
|  | Other substance abuse | Other substance abuse |
|  | Otitis media | Otitis media |
|  | Pancreatic disorders (excluding diabetes) | Pancreatic disorders (excluding diabetes) |
|  | Paralysis (other than cerebral palsy) | Paralysis (other than cerebral palsy) |
|  | Paresthesia | Skin/Subcutaneous signs and symptoms |
|  | Pericarditis and pericardial disease | Pericarditis and pericardial disease |
|  | Peripheral and visceral vascular disease | Peripheral and visceral vascular disease |
|  | Peritonitis and intra-abdominal abscess | Peritonitis and intra-abdominal abscess |
|  | Pneumonia | Pneumonia (except that caused by tuberculosis) |
|  | Pneumothorax | Pneumothorax |
|  | Polyneuropathies | Polyneuropathies |
|  | Postthrombotic syndrome and venous insufficiency/hypertension | Postthrombotic syndrome and venous insufficiency/hypertension |
|  | Pressure ulcer | Pressure ulcer of skin |
|  | Pulmonary embolism | Acute pulmonary embolism |
|  | Pulmonary heart disease | Pulmonary heart disease |
|  | Respiratory failure | Respiratory failure; insufficiency; arrest |
|  | Retinal and vitreous conditions | Retinal and vitreous conditions |
|  | Schizophrenia spectrum and other psychotic disorders | Schizophrenia spectrum and other psychotic disorders |
|  | Sedative-related disorders | Sedative-related disorders |
|  | Sequela of specified nervous system conditions | Sequela of specified nervous system conditions |
|  | Sinusitis | Sinusitis |
|  | Skin and subcutaneous tissue infections | Skin and subcutaneous tissue infections |
|  | Sleep disorders | Sleep wake disorders |
|  | Spondylopathies/spondyloarthropathy (including infective) | Spondylopathies/spondyloarthropathy (including infective) |
|  | Symptoms of mental and substance use conditions | Symptoms of mental and substance use conditions |
|  | Syncope | Syncope |
|  | Tendon and synovial disorders | Tendon and synovial disorders |
|  | Thromboembolism | Acute phlebitis; thrombophlebitis and thromboembolism |
|  | Toxic effects, subsequent encounter | Toxic effects, subsequent encounter |
|  | Transient cerebral ischemia | Transient cerebral ischemia |
|  | Trauma- and stressor-related disorders | Trauma- and stressor-related disorders |
|  | U099/B948 | PASC-General |
|  | Upper respiratory infections | Other specified upper respiratory infections |
|  | Urinary incontinence | Urinary incontinence |
|  | Vasculitis | Vasculitis |
|  | Viral infection | Viral infection |

Notes: 1. Indicates names for PASC categories defined by our clinician team after reviewing the diagnosis codes under each category and the incidence of each individual diagnosis code under each category. 2 indicates the names of the original CCSR categories.


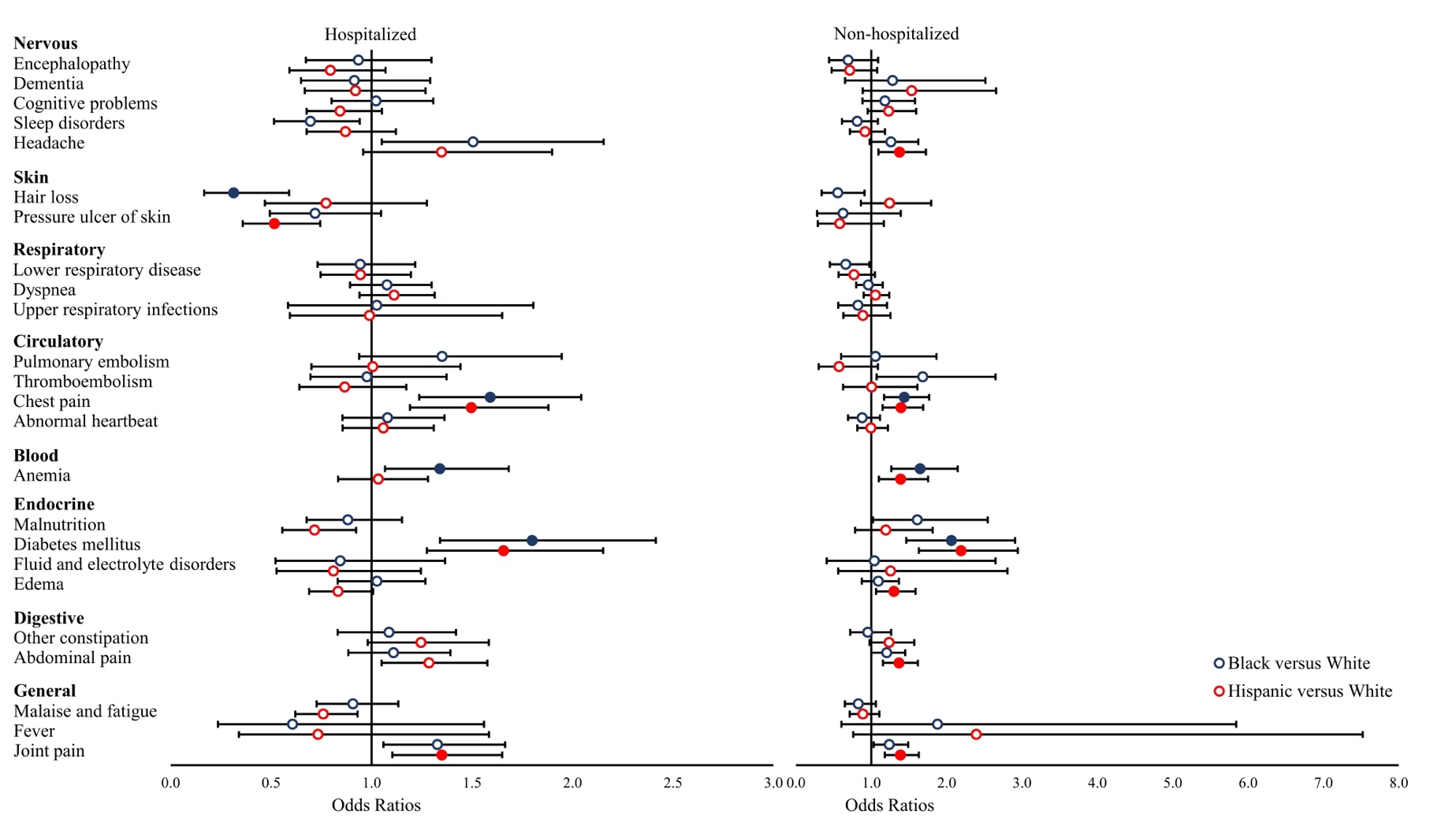
**eFigure 2. Adjusted Differences in Incidence of New Conditions and Symptoms by Race/Ethnicity Among Hospitalized and Non-Hospitalized COVID-19 Patients, Patients with a Positive PCR/Antigen Test Only**

Notes: Odds ratios (ORs) were estimated from logistic regressions examining the outcome of having at least diagnosis code of each new condition and symptom category during the follow-up period (Reference group = White). Models were adjusted for baseline patient characteristics, including age, gender, year-month of COVID-19 positive testing, comorbidities, and indicators for the five institutions contributing data. * Filled symbols indicate significant ORs that are statistically significant after false discovery rate correction (q < 0.05). Analyses only included patients with a positive PCR/antigen laboratory test (n = 27,278).

**
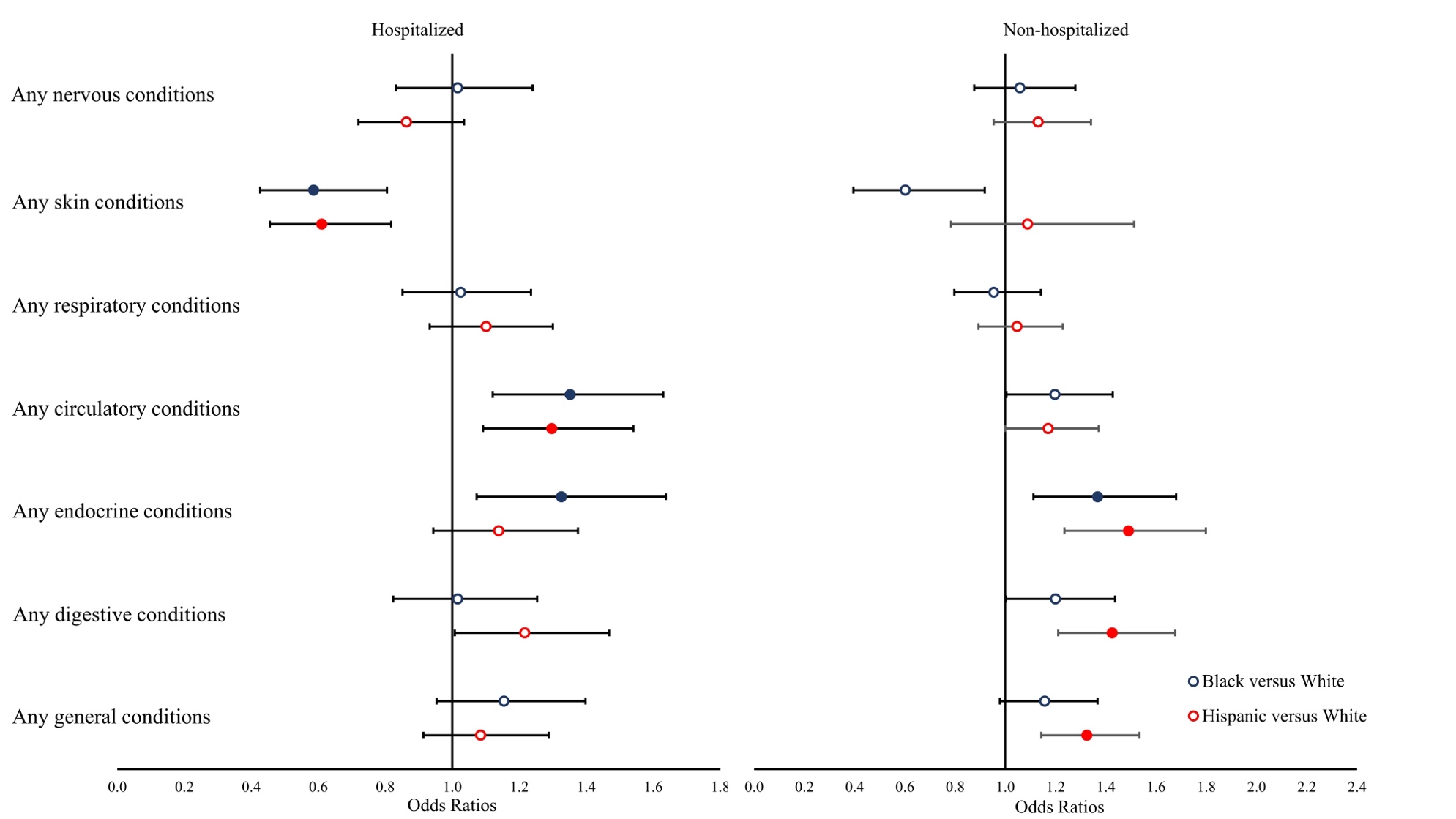
eFigure 3. Adjusted Differences in Incidence of Groups of Conditions and Symptoms by Race/Ethnicity Among Hospitalized and Non-Hospitalized COVID-19 Patients, Patients with a Positive PCR/Antigen Test Only**

Notes: Odds ratios (ORs) were estimated from logistic regressions examining the outcome of having at least one condition or symptom in each group during the follow-up period (Reference group = White). Models were adjusted for baseline patient characteristics, including age, gender, year-month of COVID-19 positive testing, comorbidities, and indicators for the five institutions contributing data. * Filled symbols indicate significant ORs that are statistically significant after false discovery rate correction (q < 0.05). Analyses only included patients with a positive PCR/antigen laboratory test (n = 27,278).

**eFigure 4. Adjusted Differences in Incidence of New Conditions and Symptoms by Race/Ethnicity Among Hospitalized and Non-Hospitalized COVID-19 Patients, Excluding COVID-19 Patients in the First Wave of the Pandemic (March through May 2020) in NYC**


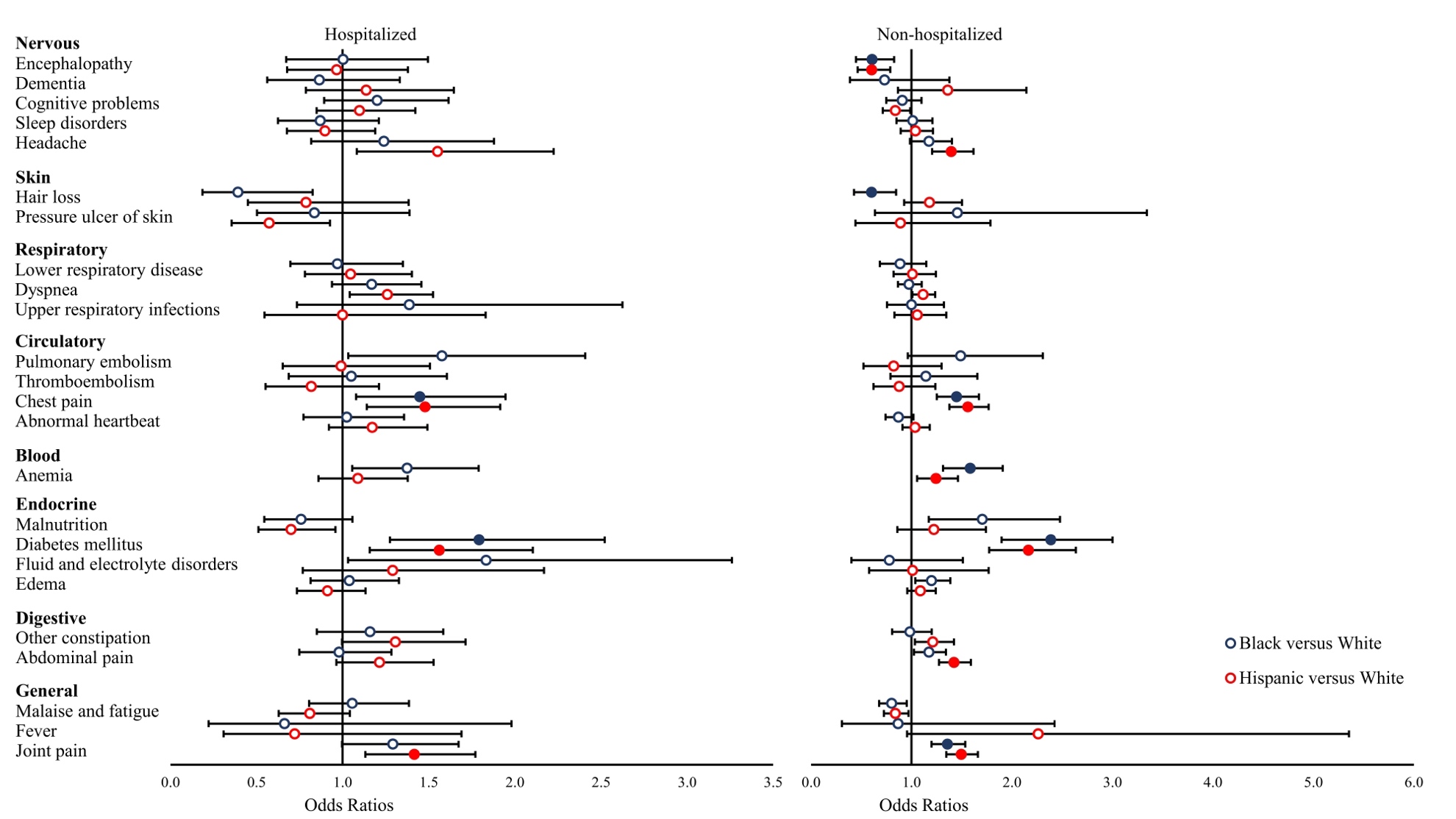


Notes: Odds ratios (ORs) were estimated from logistic regressions examining the outcome of having at least diagnosis code of each new condition and symptom category during the follow-up period (Reference group = White). Models were adjusted for baseline patient characteristics, including age, gender, year-month of COVID-19 positive testing, comorbidities, and indicators for the five institutions contributing data. * Filled symbols indicate significant ORs that are statistically significant after false discovery rate correction (q < 0.05). Analyses only included patients with COVID-19 after May 30^th^ (n = 47,476).

**eFigure 5. Adjusted Differences in Incidence of Groups of Conditions and Symptoms by Race/Ethnicity Among Hospitalized and Non-Hospitalized COVID-19 Patients, Excluding COVID-19 Patients in the First Wave of the Pandemic (March through May 2020) in NYC**


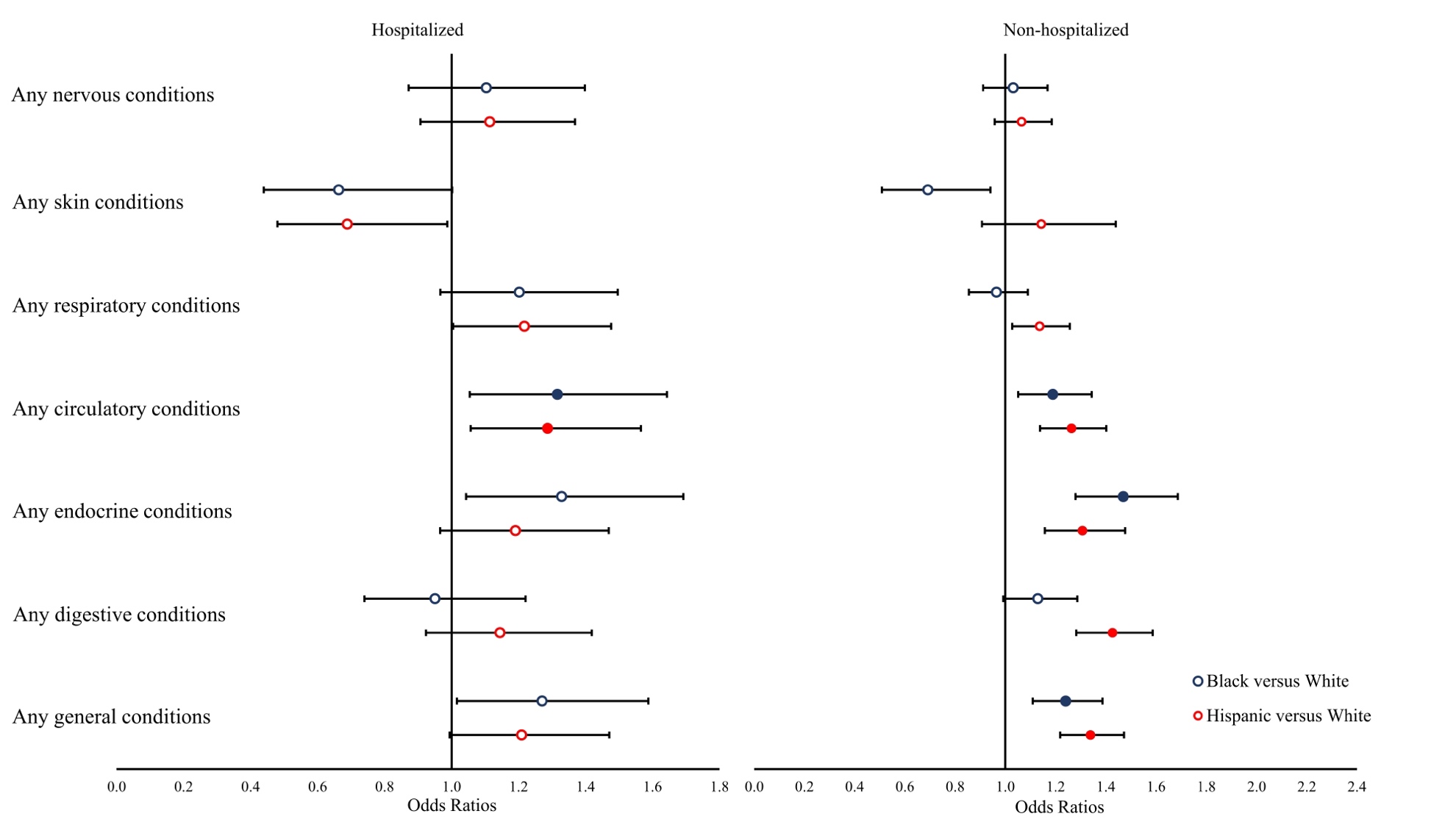


Notes: Odds ratios (ORs) were estimated from logistic regressions examining the outcome of having at least one condition or symptom in each group during the follow-up period (Reference group = White). Models were adjusted for baseline patient characteristics, including age, gender, year-month of COVID-19 positive testing, comorbidities, and indicators for the five institutions contributing data. * Filled symbols indicate significant ORs that are statistically significant after false discovery rate correction (q < 0.05). Analyses only included patients with COVID-19 after May 30^th^ (n = 47,476).

**eFigure 6. Neighborhood Social Condition Adjusted Differences in Incidence of New Conditions and Symptoms by Race/Ethnicity Among Hospitalized COVID-19 Patients**


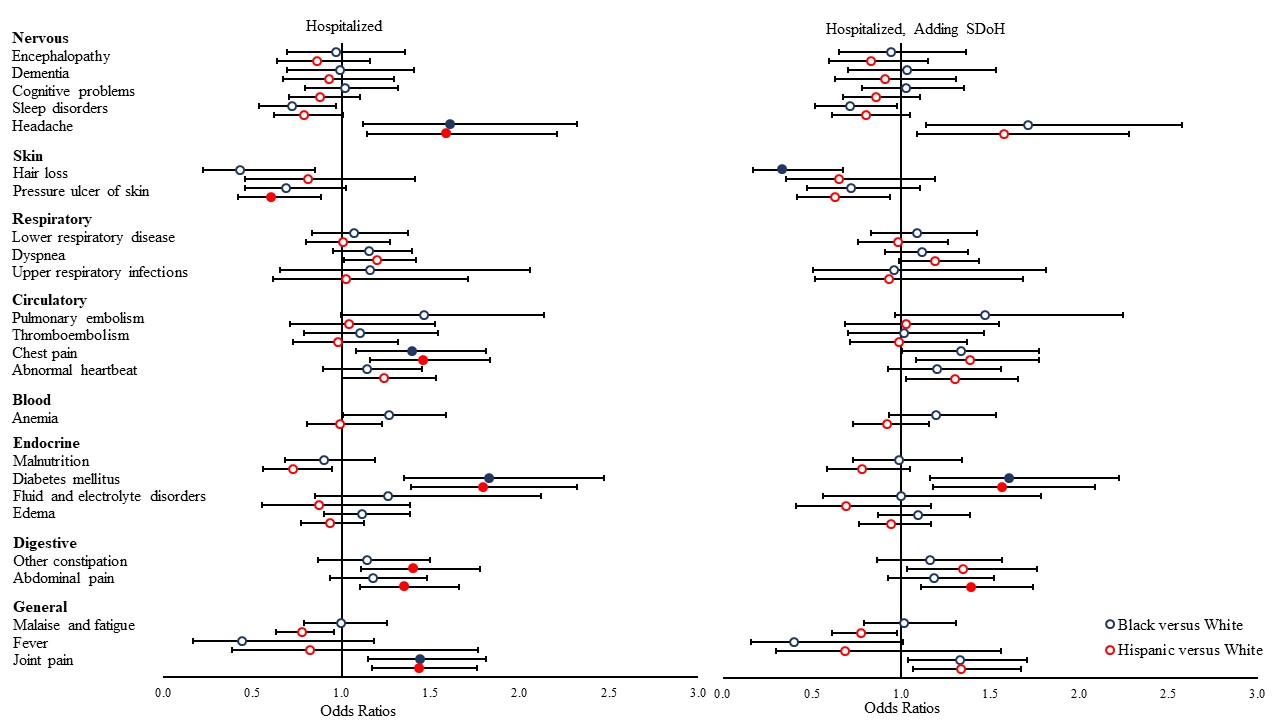


Notes: Odds ratios (ORs) were estimated from logistic regressions examining the outcome of having at least diagnosis code of each new condition and symptom category during the follow-up period (Reference group = White). Models were adjusted for baseline patient characteristics, including age, gender, year-month of COVID-19 positive testing, comorbidities, and indicators for the five institutions contributing data. Neighborhood social conditions include including median household income, unemployment rate, uninsurance rate, percent of population employed as essential workers, percent of population with limited English proficiency, percent of population foreign born, percent of population with crowded living conditions (more than 1.5 persons per room), and percent of population living in the same home for the past year, as well as the availability of neighborhood green space (as measured by the Normalized Difference Vegetation Index) from NASA’s Moderate Resolution Imaging Spectroradiometer. * Filled symbols indicate significant ORs that are statistically significant after false discovery rate correction (q < 0.05).


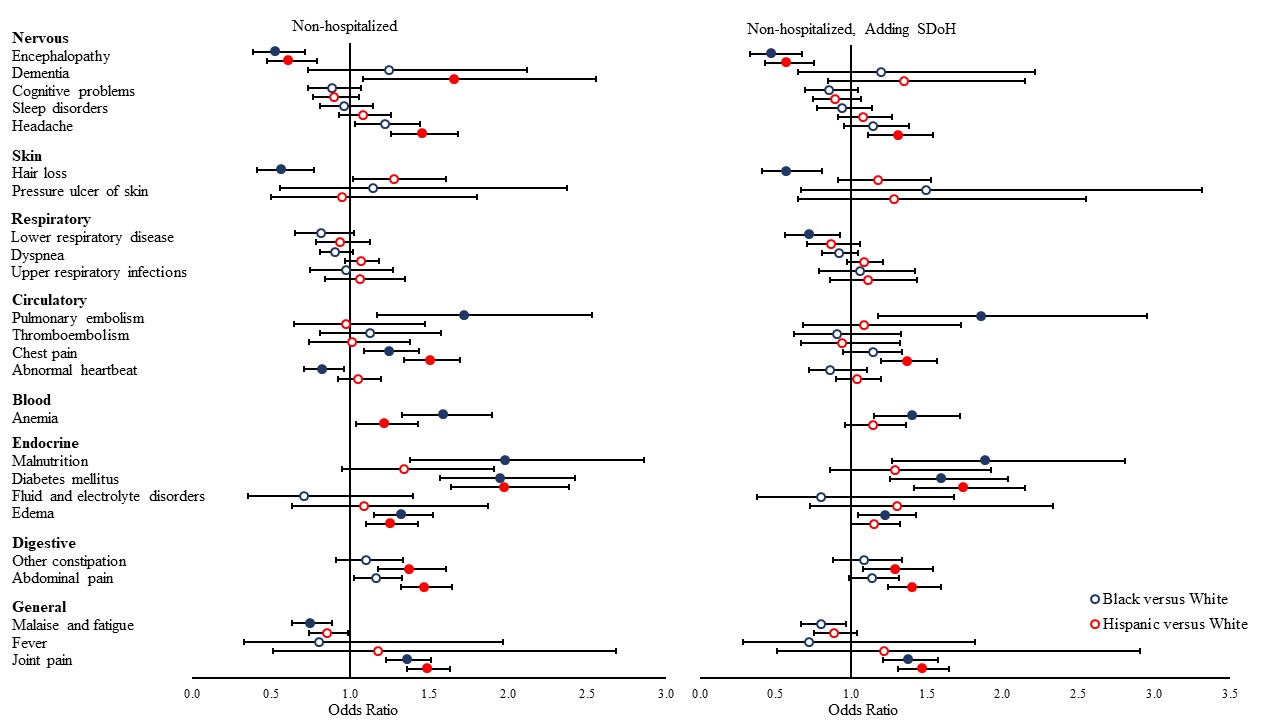
**eFigure 7. Neighborhood Social Condition Adjusted Differences in Incidence of New Conditions and Symptoms by Race/Ethnicity Among Non-Hospitalized COVID-19 Patients**

Notes: Odds ratios (ORs) were estimated from logistic regressions examining the outcome of having at least diagnosis code of each new condition and symptom category during the follow-up period (Reference group = White). Models were adjusted for baseline patient characteristics, including age, gender, year-month of COVID-19 positive testing, comorbidities, and indicators for the five institutions contributing data. Neighborhood social conditions include including median household income, unemployment rate, uninsurance rate, percent of population employed as essential workers, percent of population with limited English proficiency, percent of population foreign born, percent of population with crowded living conditions (more than 1.5 persons per room), and percent of population living in the same home for the past year, as well as the availability of neighborhood green space (as measured by the Normalized Difference Vegetation Index) from NASA’s Moderate Resolution Imaging Spectroradiometer. * Filled symbols indicate significant ORs that are statistically significant after false discovery rate correction (q < 0.05).
